# The negative impact of wearing personal protective equipment on communication during coronavirus disease 2019

**DOI:** 10.1017/S0022215120001437

**Published:** 2020-07-09

**Authors:** T Hampton, R Crunkhorn, N Lowe, J Bhat, E Hogg, W Afifi, S De, I Street, R Sharma, M Krishnan, R Clarke, S Dasgupta, S Ratnayake, S Sharma

**Affiliations:** Departments of Ear Nose and Throat Surgery and Audiovestibular Medicine & Audiology, Alder Hey Children's Hospital NHS Foundation Trust, Liverpool, UK

**Keywords:** Communication, Protective Devices, Coronavirus Infections, Health Personnel, Noise

## Abstract

**Background:**

Coronavirus disease 2019 personal protective equipment has been reported to affect communication in healthcare settings. This study sought to identify those challenges experimentally.

**Method:**

Bamford–Kowal–Bench speech discrimination in noise performance of healthcare workers was tested under simulated background noise conditions from a variety of hospital environments. Candidates were assessed for ability to interpret speech with and without personal protective equipment, with both normal speech and raised voice.

**Results:**

There was a significant difference in speech discrimination scores between normal and personal protective equipment wearing subjects in operating theatre simulated background noise levels (70 dB).

**Conclusion:**

Wearing personal protective equipment can impact communication in healthcare environments. Efforts should be made to remind staff about this burden and to seek alternative communication paradigms, particularly in operating theatre environments.

## Introduction

During the coronavirus disease 2019 (Covid-19) pandemic, when supplies have been sufficient, healthcare professionals worldwide have delivered care to their patients whilst wearing mandated personal protective equipment (PPE).^1^ The authors anecdotally found communication and understanding when wearing PPE to be drastically reduced in clinical areas. This impact of PPE in general on communication has previously been raised in popular press^2^ and scientific literature.^3–5^ We sought to experimentally assess these difficulties through a simulated clinical environment model.

In the clinical context, workers frequently speak and communicate in the presence of background noise, rather than the ‘gold standard’ silence of an audiological testing booth. In day-to-day hearing assessment, pure tone audiometry represents the gold standard test for hearing ability and is a good measure of hearing impairment. However, the audiogram generated by the pure tone audiometry is a poor indicator of speech recognition in noise.^6^ Pure tone audiometry measures hearing sensitivity, rather than assessing the auditory and speech processing ability of the subject; therefore, findings from pure tone audiometry do not always correlate with the functional hearing ability of subjects faced with real-world signals and noise, such as speech.^7^ A words-in-noise task adds significant cognitive load versus the same task without noise. In clinical settings, there will always be a degree of background noise; hence, a speech-in-noise test was felt to be a better real-world ‘stress test’ of auditory function.^8^

Rather than a test of hearing, speech-in-noise testing for adults can assist clinicians in assessing a patient's speech understanding in noise. Screening tests that use sentences rather than single words or phonemes are now preferred to monosyllabic word lists in quiet conditions, as it has been demonstrated that these single word lists have limited reliability and lack validity in relation to real-world simulations.^9–13^ On this basis, we sought to identify if there were genuine measurable challenges to speech discrimination whilst wearing Covid-19 PPE by using speech-in-noise tests.

## Materials and methods

We sought to reproduce the background noise levels experienced by clinicians, by adjusting the signal-to-noise ratio during testing. We chose adaptive signal-to-noise ratio, using Bamford–Kowal–Bench sentence lists read by a clinician^14^ whilst a Parrot machine (Parrotplus 2; Soundbyte Solutions, Leigh, UK)^15^ produced the background babble noise (simulated speech such as you might hear in a crowded pub or emergency department) at predetermined levels of noise. The Parrot machine is a portable, digital speech screening system for assessing speech discrimination using a range of recognised speech discrimination tests.

The Bamford–Kowal–Bench sentences used in this test were published in 1979 as a protocol for testing hearing impaired children, and were developed as a speech-in-noise test by Niquette *et al*., in 2003.^16^ There are 10 sentences in each list, and 18 lists in total to prevent repetition. Each sentence has three or four words that must be repeated by the subject. A percentage score can be given for how many key words are correctly repeated.

In order to determine the background noise levels in our hospital, we conducted two 30-second sound meter recordings (using an ATP® SL-8928 digital sound level meter (calibrated by National Health Service audiometric calibration service, Audiology Department, Withington Community Hospital, Manchester)) in four discrete environments, all during normal, daylight working hours; namely, the office, the emergency department, the intensive care unit and the operating theatre.

The minimum and maximum background noise levels (during daylight hours, with regular levels of staff) were recorded as follows: 40–55 dB for the office, 48–66 dB for the emergency department, 50–78 dB for the intensive care unit and 53–84 dB for the operating theatre.

Five candidates representing our hospital ENT department were selected for participation, comprising two women and three men. Their age range was 29–49 years, with a median age of 39 years.

Initial 0.25–8 kHz pure tone audiograms were conducted to confirm no significant hearing loss in our five candidates, who had no previous otological history or significant co-morbidity.

All testing was conducted in a soundproofed audiometry booth. The baseline standard Bamford–Kowal–Bench sentence test was conducted in silence for all candidates, without PPE. Scores were 100 per cent for all candidates.

We then conducted the Bamford–Kowal–Bench sentence test whilst each subject wore the facial PPE suitable for aerosol-generating procedures (fit-tested filtering facepiece code 3 mask and head visor). All subjects had previously undergone fit testing to ensure that the PPE worn fitted appropriately for each individual.

The researcher read the Bamford–Kowal–Bench word lists whilst wearing aerosol-generating procedure PPE. The subject wore the same PPE at a distance of 2 m. The Parrot machine was placed behind and above the head of the researcher. Background noise (adult) babble settings were chosen to represent different environments as follows: 45 dB for the office, 55 dB for the emergency department, 65 dB for the intensive care unit and 70 dB for the operating theatre.

Each candidate underwent Bamford–Kowal–Bench testing at the four background noise levels, in three test conditions: (1) candidate and researcher in normal conditions without PPE, with the researcher's voice at normal volume levels; (2) candidate and researcher both in aerosol-generating procedure PPE, with the researcher's voice at normal volume levels; and (3) candidate and researcher both in aerosol-generating procedure PPE, with the researcher attempting to raise their voice. A raised voice reflected an increase in voice volume to the point at which the researcher felt their voice was comprehensible against the background noise.

The percentage of key words in the Bamford–Kowal–Bench sentences repeated by the candidate was recorded. Each sentence was read once by the researcher and was not repeated.

During day-to-day working and conversation, people do not speak at the same intensity throughout a whole conversation. Background noise also fluctuates, rather than remaining at constant levels. Hence, we decided to use a live, fluctuating voice, rather than pre-recorded voices amplified to a fixed and constant volume.^17^ When speakers adjust their voice to overcome background noise, this is known as the Lombard effect. Although attempting to raise one's voice or shout usually causes only a small increase in volume between 5 dB and 10 dB,^18^ we chose to measure the volume of voice produced by the researcher as a secondary outcome measure. This was not our primary concern, as attempts to raise one's voice in day-to-day clinical practice will have both inter- and intra-person variability. Hence, we felt that the simulation integrity was preserved, regardless of actual volume levels produced by the researcher.

### Statistical analysis

The primary outcome measures were: differences in Bamford–Kowal–Bench sentence test results in various stimulated environments (office, emergency department, intensive care unit and operating theatre); and differences in Bamford–Kowal–Bench sentence test results in various PPE equipment scenarios (no PPE, wearing PPE, and wearing PPE whilst raising voice volume).

The secondary endpoints were: measurement of mean change in voice volume in response to an increase in background noise; and mean signal-to-noise ratios with different PPE equipment scenarios and environments.

Data were analysed using IBM SPSS Statistics (IBM, Armonk, New York, USA). Differences in Bamford–Kowal–Bench scores for the various PPE equipment simulations in each hospital environment were calculated using one-way repeated analysis of variance (ANOVA) tests. For any environment found to have a statistically significant result, further comparison of the differences in Bamford–Kowal–Bench sentence test results with different PPE equipment scenarios were analysed using the Wilcoxon signed-rank test. A *p*-value of less than 0.05 was considered statistically significant. The Wilcoxon signed-rank test was used as it compares dependant rather than independent samples.

### Patient and public involvement

This research was conducted without patient involvement. Patients were not consulted to develop outcomes or interpret the results, as the focus was staff communication. The public may be involved in future, particularly individuals who are deaf or hard of hearing, if this research is expanded to include clinician–patient communication.

## Results

Table 1 presents the Bamford–Kowal–Bench sentence test results for the five people entered into the study.

**Table 1.** Bamford–Kowal–Bench sentence test results

Subject number	Office			Emergency department			Intensive therapy unit			Operating theatre		
	No PPE	PPE	PPE shout*	No PPE	PPE	PPE shout*	No PPE	PPE	PPE shout*	No PPE	PPE	PPE shout*
1	100	92	100	100	88	100	98	88	92	94	58	86
2	96	98	98	98	90	100	88	78	80	84	54	74
3	98	100	100	100	96	94	100	76	94	94	62	88
4	100	84	100	70	92	96	100	60	88	74	54	80
5	100	100	100	100	100	100	100	96	100	92	90	100

Data represent Bamford–Kowal–Bench sentence test scores (percentages). *Raised voice whilst wearing personal protective equipment. PPE = personal protective equipment

One-way repeated-measures ANOVA indicated that different PPE equipment scenarios did not significantly alter Bamford–Kowal–Bench sentence test results in office or emergency department settings (*p* = 0.26 and *p* = 0.58 respectively), but showed a trend in intensive care unit settings (*p* = 0.06). The statistical assumption of sphericity in the intensive care unit setting was marginally violated, an effect perhaps due to the small sample size. If sphericity was assumed, the one-way repeated measured ANOVA test was statistically significant (F(2,8) = 6.64, *p* = 0.02, ηp2 = 0.73). The assumption of sphericity was not violated in the operating theatre setting results (χ^2^ (2) = 3.13, *p* = 0.21), and different PPE equipment scenarios significantly altered Bamford–Kowal–Bench sentence test results (F(2,8) = 17.16, *p* = 0.001, ηp2 = 0.81).

A Wilcoxon signed-rank test indicated that Bamford–Kowal–Bench sentence test scores were significantly lower for subjects wearing PPE (median score = 58) compared to those without PPE (median score = 92) in an operating theatre simulated environment (Z = −2.02, *p* = 0.04). Increasing voice volume whilst wearing PPE significantly increased Bamford–Kowal–Bench sentence test scores (median score = 86) compared to normal speech volume when wearing PPE (median score = 58; Z = 2.03, *p* = 0.04). There was no significant difference in Bamford–Kowal–Bench scores when wearing no PPE (median score = 92) compared to when raising voice volume whilst wearing PPE (median score = 86) (Z = −0.68, *p* = 0.50).

• This novel study experimentally addresses hearing and communication difficulties currently experienced by healthcare personnel during coronavirus disease 2019 pandemic
• Speech discrimination scores were significantly different between normal and personal protective equipment (PPE) wearing subjects in operating theatre simulated background noise levels (70 dB)
• Performance was also worse in simulated intensive care unit noise levels
• Wearing PPE can impact communication, which has implications for patient safety
• Staff should be reminded of this burden and alternative communication paradigms sought, particularly in operating theatre environments

Our secondary outcome measure was mean change in voice volume when wearing PPE. The increase in background noise rose by 25 dB, from 45 dB (simulated office) to 70 dB (simulated operating theatre). Our researcher elevated their natural voice by 13–20 dB without PPE in response to simulated increasing sound levels. This correlates with existing studies showing a natural shift to maintain signal-to-noise ratio in human speech.^18^

Mean voice volumes across all simulations tended to increase with PPE wearing, and increased again with PPE wearing and raised voice. The mean signal-to-noise ratios measured are shown in Table 2.

**Table 2.** Mean signal-to-noise ratio results

Parameter	Office			Emergency department			Intensive therapy unit			Operating theatre		
	No PPE	PPE	PPE shout*	No PPE	PPE	PPE shout*	No PPE	PPE	PPE shout*	No PPE	PPE	PPE shout*
Background noise (dB)	45	45	45	55	55	55	65	65	65	70	70	70
Mean SNR (dB)	+2.9	+4.7	+16.4	−3.2	+4.1	+7.6	−5.8	−4.9	+2	−7.4	−7.4	+0.2

* Raised voice whilst wearing personal protective equipment. PPE = normal voice with personal protective equipment; SNR = signal-to-noise ratio

## Discussion

Our study findings support our assumption that wearing facial aerosol-generating procedure PPE reduces staff understanding and conventional communication in simulated intensive care unit and operating theatre settings. Despite the small sample size, the results suggest that the louder background environments, such as an operating theatre setting, produced the most pronounced (statistically significant) effect on speech comprehension. This could have a significant impact on patient safety.

The excess noise generated in such environments can be attributed to many factors (aptly summarised in the paper by Kam *et al*.^19^), ranging from equipment and type of surgical or anaesthetic activity, to numbers of personnel and consequent raised voice levels. We have demonstrated that wearing PPE will complicate communication further. In this study, speech comprehension whilst wearing PPE within the operating theatre simulated environment (70 dB) was significantly worse than whilst wearing no PPE. The raising of voice in an operating theatre simulated environment when wearing PPE caused a significant improvement of Bamford–Kowal–Bench scores to a level that was not significantly different from scores when not wearing PPE.

Despite the observed variance in signal-to-noise ratio, Bamford–Kowal–Bench scores were still generally poorer with PPE, which may indicate a difficulty in understanding that is unrelated to volume or signal-to-noise ratio, but is instead related to loss of expressions or lip-reading.

Background levels of noise in our simulation were derived from environmental recordings that correlated with prior studies, where noise levels in the operating theatre exceed World Health Organization (WHO) recommendations.^20^ It has been suggested that noise masking speech in the operating theatre often results in surgeons having to repeat themselves; consequently, it takes longer for other members of the team to respond or assist.^20^ Previous research has investigated background noise and its impact on staff adherence to the WHO surgical safety checklist, but outside of calls for quiet during the ‘time out’ phase of an operation, perceptions of barriers to communication during the rest of the procedure are less well investigated.^21^

We have confirmed anecdotal reports that communication difficulties due to PPE will impact significantly on healthcare workers. The safety of patients and healthcare staff is paramount, and the ongoing use of PPE as the initial Covid-19 pandemic wanes is likely to continue. Therefore, we anticipate that these communication issues will be exacerbated, particularly in operating theatres, where anaesthetists, operating department practitioners, nurses and surgeons will wear aerosol-generating procedure PPE for prolonged durations as some longer procedures and elective operating recommence.

When interpreting our findings, it is important to consider that we simulated background noise, and the study candidates were healthcare staff without hearing impairment, who regularly work together, which may mean that our results would not be reproduced ‘in the field’. Clinical situations regularly involve a variety of shift-working healthcare providers and the additional cognitive load of actually treating patients, all of which could further hinder communication. Nonetheless, our simulation used validated speech testing, and we are unaware of any other studies that have assessed communication difficulties with Covid-19 PPE to this standard.

The importance of speech understanding for achieving success on shared objectives has been extensively researched in military and industrial-occupational settings, with a need to communicate with co-workers in noisy backgrounds regularly resulting in the removal of protective equipment.^22^ There are obvious and immediate implications in the operating theatre, such as compromised safety, wrong instrument selection or inadequate delivery of the WHO checklist.

Studies have suggested that as much as 12 dB signal-to-noise ratio is required for speech understanding in the presence of background noise levels up to 110 dB SPL,^23^ but thresholds for adults have also been recorded with ratios close to 0 dB or less than 0 dB.^24^

One impact of PPE we have not investigated is the removal of visual cues to communication. Various studies have demonstrated that visual features strongly affect the perception of speech.^17^ This contribution is most pronounced in noisy environments, where the intelligibility of audio-only speech is quickly degraded.^25^

We recommend that regular reminders to speak up and acknowledge communication difficulties at key times during intensive care unit ward rounds and operating theatre pre-surgery briefs may help staff to improve communication whilst wearing PPE. Some specialty guidelines have recommended staff members wearing photographs of themselves over their PPE, or writing their name and roles on the apron.^26^ Some centres have advocated communicating with hand signals, transparent masks or hoods,^17^ using white boards, or even employing a two-way radio or walkie talkies in cellophane bags.^27^ Others have suggested possible wireless microphone and speaker systems incorporated into PPE (Micrashell PPE suit^28^), or even currently used PPE designs with voice amplification solutions^29^ that utilise mobile phone technology. For modern multidisciplinary teams, this may not be a suitable solution when there needs to be multidirectional conversation and information exchange. Solutions like Cardmedic (a free-to-use collection of communication flashcards) have been designed to help healthcare workers speak to patients despite PPE,^30^ but we are unaware of any similar device specific to communication between healthcare workers in settings such as the operating theatre.

The primary drawback in this study was the small sample size, which did not allow us to measure effect size. In addition, the study was performed in one hospital site only, and representative environmental noise levels could differ across different hospital sites. We welcome the opportunity to work with other teams across the UK and further afield in testing, trialling and simulation, as well as supporting qualitative work for any PPE communication solutions for future working practices.

## Conclusion

Where attempts to deliberately raise voice volume or shout through PPE were simulated, understanding significantly improved as expected. The raising of voice for prolonged periods may lead to issues with voice strain and abuse, in addition to frustration or miscommunication. We hope that now communication difficulties with PPE have been scientifically demonstrated, this will help to drive attempts to mitigate these issues for healthcare workers when emerging from the Covid-19 pandemic. We hope our findings can inform the ongoing use of PPE as elective healthcare provision is restarted, and for the future, facing whatever pandemics may lie ahead.
